# Validity of STONE Score in Clinical Prediction of Ureteral Stone Disease

**DOI:** 10.12669/pjms.36.7.2625

**Published:** 2020

**Authors:** Abdul Malik, Syed Mohkumuddin, Shazia Yousaf, Mirza Ahmad Raza Baig, Ayesha Afzal

**Affiliations:** 1 Dr. Abdul Malik FCPS Medicine, FCPS (Fellow Nephrology), Senior Registrar Internal Medicine, Sandeman Provincial Hospital, Quetta, Pakistan; 2 Dr. Syed Mohkumuddin (FCPS Nephrology), Assistant Professor Nephrology, Sandeman Provincial Hospital, Quetta, Pakistan; 3 Dr. Shazia Yousaf (FCPS Medicine), Senior Registrar, Medical Unit, Sandeman Provincial Hospital, Quetta, Pakistan; 4 Mr. Mirza Ahmad Raza Baig. Clinical Perfusion Specialist, Hail Cardiac Center, Saudi Arabia.; 5 Miss. Ayesha Afzal, University of Health Sciences Lahore, Lahore, Pakistan

**Keywords:** STONE score, Ureteral stones

## Abstract

**Objective:**

To determine the external validity of STONE score for predicting the probability of ureteral stone in patients presenting in emergency department with suspicion of ureteral stones.

**Methods:**

In this prospective validation study, a total of 134 patients aged above 18 years, and first time arrived in the emergency unit for treatment of flank pain and then referred for the CT scan for suspected ureteral stone in Sandeman Provincial Hospital, Quetta, from 10-June-2018 to 15-Oct-2019 were included. STONE score calculation was done before sending the patient to the CT scan, using the same protocol as defined by Moore et al. Based on STONE score patients classified into the low-risk group (0 to 5), moderate-risk group (5 to 9) and the high-risk group (10 to13). The AUC, sensitivity, specificity and test characteristics were calculated for STONE score.

**Results:**

The mean age was 39.2± 11.2 years, there were 86 (64.17%) men and 48 (35.83%) women. there were 26.8% patients having low-risk score, 52.23% moderate-risk and 21.97% high-risk score. On receiver operating curve (ROC) the area under curve (AUC) of the stone score was 0.75 (95% CI, 0.67 to 0.83), the lower band of AUC 0.67 and upper band 0.83. In high risk STONE score the sensitivity of STONE score was 66.7% and specificity was 75.0%.

**Conclusion:**

Based on our study results, CT scan and ultrasonography are standard diagnostic tools for suspected ureterolithiasis but in emergency unit, use of STONE score to categorize the patient as low risk, moderate-risk and high-risk of ureteral stone can help the physician (clinician) to take decision either there is a need of further investigation or not.

## INTRODUCTION

Ureterolithiasis (ureteral stone) is a common disease that has an unusual impact on quality of life. The pace of ureteral stone patients rising around the world regardless of age and gender.^1^ Individuals with ureterolithiasis express acute pain, the pain occurs due to unable to pass stones in urine.^2^ Patients with flank pain visit the emergency unit and 90% of patients are discharged with assessment and symptomatic therapy, remaining treated by hospital admission.^3^ Admitted patients eventually undergo urologic intervention. calcium oxalate (75% to 90%) is a vital component of kidney stones. the prevalence rate of the nephrolithiasis from 1% to 5% in Asia^4^, 16 % in Pakistan (7.4% in north Pakistan and 28% in west of Pakistan).^5^ Diabetes, hypertension and obesity are common risk factors of urolithiasis in Pakistan.^6^

In recent times, clinicians improved competency in diagnosis and management of ureter stones, individuals suspected of ureteral stones in the emergency unit, Clinicians use STONE score to predict of ureteral risk without depending on further imaging. STONE score was developed by Moore et al.^2^ and was established in Yale University Medical School, the calculation of STONE score is depending on the total of five categorical predictors (male gender, pain duration, nausea/vomiting, non-black race and Red blood cells on urine dipstick), each predictor point is calculating on the estimated coefficient from a regression to predict the presence of a ureteral stone. The score ranges from 0 to 13. Based on STONE score patients classified into the low-risk group (0 to 5), moderate-risk group (5 to 9) and the high-risk group (10 to13).^7^ According to research data, the high STONE score may undergo ultrasonography and CT scan for further investigation,^8^,^9^ but still there is a lack of clear guidelines regarding STONE score. And only limited number of studies is there for validation of STONE score in predicting urolithiasis so there is a need to verify the strength of STONE score in more races. Like in Pakistan there is no black race so three points are added to each of our patients. So, in present study we determined the external validity of STONE score for predicting the probability of ureteral stone in Pakistani patients presenting in emergency department with suspicion of ureteral stones.

## METHODS

In this prospective study, all the patients who were admitted to the emergency unit of Sandeman Provincial hospital Quetta, with flank pain, from 10-June-2018 to 15-Oct-2019 were included. The study inclusion criteria were; age above 18 years, the first time arrived in the emergency unit for treatment of flank pain then referred for the CT scan for suspected ureteral stone. Exclusion criteria were patients under urological treatment, referred from other hospitals for Nephrolithiasis treatment and finally incomplete records (microscopic hematuria). A total number of 146 patients were included during the study period. Due to the absence of incomplete records (microscopic hematuria) we omitted 12 patients from the study, the final count was 134 patients. The current study approved by the institutional ethical administrative committee (Ref # 5717, dated; 09-April-2020). Before the data collection, written and informed consent obtained from all patients. We ensured the patient, that his data and identity never disclosed at any circumstances.

STONE score calculation was done before sending the patient to the CT scan, using the same protocol as defined by Moore et al. (Yale University of Medical school) followed for scoring, all patients’ data were collected as per the STONE score protocol (Table-I) such as 1.sex 2. duration of pain 3. Nausea/vomiting occurrence 4. race (Non-black/black) and 5. Microscopic hematuria (RBCs) in the urine. The score ranges from 0 to 13. Based on STONE score patients classified into the low-risk group (0 to 5), moderate-risk group (5 to 9) and the high-risk group (10 to13).^7^

**Table-I:** Moore’s STONE score table.

Category	Characteristic	Score
Gender	Male	2
	Female	0
Pain duration	< 6 hours	3
	6 to 24 hours	1
	>24 hours	0
Race	Black	0
	Non-black	3
Nausea and vomiting	No	0
	Only nausea	1
	vomiting	2
Microscopic hematuria	Yes	3
	No	0

Total		13

Data analysis was carried out by SPSS v25.0 (IBM corp., USA), quantitative variables were calculated as Mean ± SD, and Categorical variables like STONE score were calculated as frequency and percentage. ROC curve was formulated to calculate area under the curve (AUC) for calculation of diagnostic accuracy of STONE score.

## RESULTS

A total of 134 patients met the inclusion criteria are identified for the study, the mean age was 39.2±11.2 years, there were 86 (64.17%) men and 48 (35.83%) women. The duration of pain < 6 hours. was in 30 (22.38%) patients, 6 to 24 hrs. in 41 (30.59%) and >24 hours in 63 (47.01%) patients. There were no black race individuals, there was 70 (52.23%) patients had no symptoms of nausea and vomiting, only nausea reported in 38 (28.35%) patients and vomiting occurred in 26 (19.40%) patients. Microscopic hematuria (erythrocytes) in urine was present in 92(68.65%) and absent in 42(31.35%) patients (Table-II).

**Table-II:** Characteristic of patients in ER for suspected of ureter stones.

Category	Characteristic	Ideal STONE score points	Patients (%) n=134
Gender	Male	2	86(64.17%)
	Female	0	48(35.83%)
Pain duration	< 6 hrs.	3	30(22.38%)
	6 to 24 hrs.	1	41(30.59%)
	>24 hrs.	0	63(47.01%)
Race	Black	0	0(0%)
	Non-black	3	134(100%)
Nausea and vomiting	No	0	70(52.23%)
	Only nausea	1	38(28.35%)
	vomiting	2	26(19.40%)
Microscopic hematuria	Yes	3	92(68.65%)
	No	0	42(31.35%)
		13	

Distribution of STONE score risk, there were 26.8% patients having low-risk score, 52.23% moderate-risk and 21.97% high-risk score. The diagnostic finding of Nephrolithiasis by STONE score risk category calculated based on Moore LC points, 16.66% in a low-risk group, 54.28% in a moderate-risk group and 85.71% in the high-risk group. (Table-III) according to Moore’s original study suggested that the patients with high score (high risk group) could able to undergo for the further diagnostic test like ultrasonography and low dose CT scan for confirmation of ureterolithiasis.

**Table-III:** External validation of STONE score distribution and risk category diagnosed with ureteral stone.

STONE score risk category	STONE score distribution n (%)	Ureteral stone validating with STONE score
Low risk (0 to 5 points)	36(26.86%)	16.66%
Moderate-risk (6 to 9 points)	70(52.23%)	54.28%
High-risk (10 to 13 points)	28(21.97%)	85.71%

**Fig.1:**
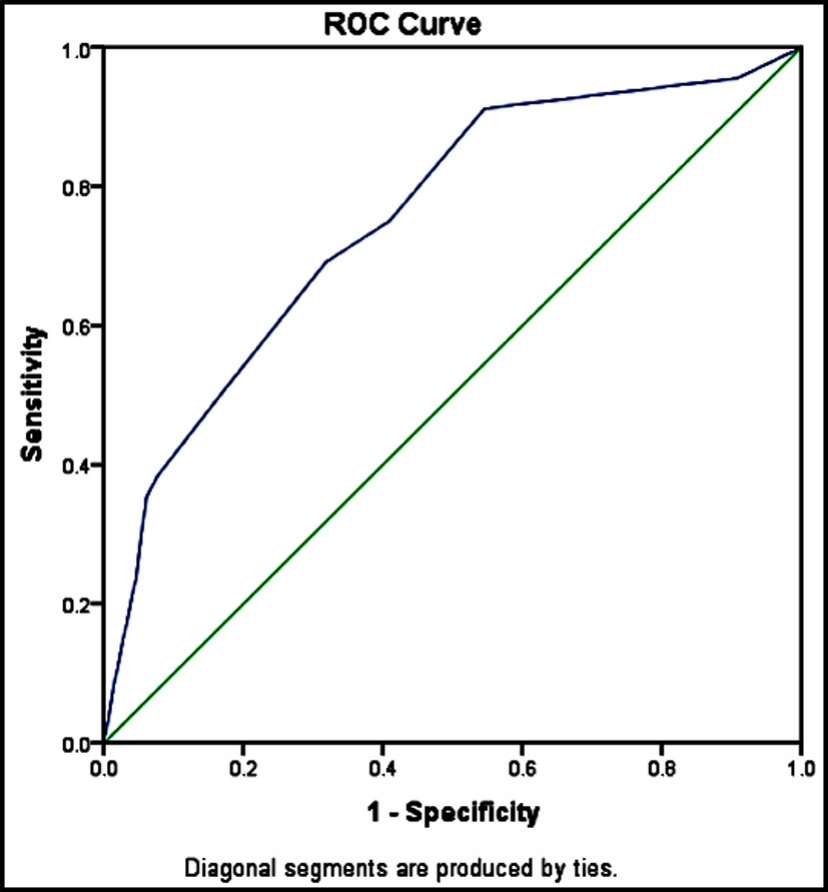
Receiver operative curve of the STONE Score.

The receiver operating characteristic (ROC) curve of the STONE score (0-13) was presented in the (Fig.1), the ROC curve looks towards to the left upper corner. The area under curve (AUC) of the stone score was 0.75 (95% CI, 0.67 to 0.83), the lower band of AUC 0.67 and upper band 0.83, it has been recommended that AUC between 0.70 to 0.80 could be fair and acceptable consideration, (AUC 0.8 to 0.9 consider as an excellent).^10^ According to our statistical results, the coordinates of the curve shows if STONE score greater than 6.5, the sensitivity is 0.75 and 1-specificity 0.40. ROC curve indicating STONE score is acceptable fit for ureterolithiasis. In high risk STONE score the sensitivity of STONE score was 66.7% and specificity was 75.0%.

## DISCUSSION

CT scan has been recommended as the best diagnostic test for finding ureterolithiasis.^11^ CT scan also enables clinicians to find alternate diagnosis like appendicitis and diverticulitis in flank pain. Keeping in vies the above mentioned benefits the usage of CT scan in ureter pain patients increased more than 10 folds from 1997 to 2007 in patients of flank pain but it did not brought any major improvements in outcomes.^12^-^14^ CT scan also lead to radiation exposure, length of hospital stay and is also a costly one.^15^,^16^

According to European Urology Association 2011 guidelines, ultrasonography should be the first-line diagnostic for the ureterolithiasis.^2^ If every flank pain patient in emergency unit undergo for CT scan to find out the kidney stone, there should be one million of CT scans has to be done every year in Pakistan, which releases a lot of ionizing radiation that may lead to malignancy. Before the introduction of STONE score, there was a study from the intravenous pyelography, they found some common factors from 73 patients with four predictive elements to identify ureteral stone: flank pain, microscopic hematuria, acute pain at onset, and radiographically positive.^17^ the present study validates Moore’s STONE score to reliable tool to find out the ureteral stones in suspected nephrolithiasis. The STONE scoring system evaluates the total points of five objectives that present at emergency unit admission (sex, duration of pain, nausea and vomiting, microscopic hematuria and race). The STONE tool is user-friendly to the emergency unit physicians to counseling patients about suspected ureter stone, further diagnosis, and treatment. Present study results of the STONE score for ureter stone were mimic Moore’s internal validation.

The study we conducted do not have any black patients (race) but in Moore’s study, there were 10 to 11 % of black patients. The calculation of race points different from country to country. Our study results provide one more strong positive evidence of the STONE score. the age and sex distribution of our study almost the same in comparison to Moore’s internal validation score.

Another study by Wang et al. validated the diagnostic accuracy of stone score, the authors reported STONE score 10-13 has a sensitivity of 53%, and specificity 87%, stone score 11-13 is 37% sensitive and 92% specific, while stone score 5-13 is 96% sensitive and 23% specific. The authors reported that STONE score is a valuable tool to stratify patients into low, middle and high risk of having ureteral stones.^18^

Although STONE score is a good predictor but it has some limitations such as presence of non-black race is three points and in many countries such as Pakistan where black race is very rare everybody gets these three points. In our suggestion, there is a need to modify STONE score regarding this issue, perhaps it will help to attain higher diagnostic accuracy of STONE score in white populations. As some authors have tried to improve the accuracy of STONE score, Daniels et al., added the presence of hydronephrosis on ultrasonography in STONE score and the specificity of score increased to 98% in low and moderate risk patients. However, the accuracy was unchanged for high risk STONE score patients.^19^ Kim et al., also modified the STONE score, they added CRP levels at cut-off 0.5 and removed race, nausea and vomiting, the AUC of STONE score after modification increased to 0.94 from 0.92 and sensitivity increased to 80% as compared to only 54% before modification.^20^

The improvement in the diagnosis of ureter stone in the emergency unit, the STONE score is useful for optimal pain control and imaging studies. Pain management in suspected ureterolithiasis will improve the quality of care and decrease the usage of analgesic drugs.^21^ as mentioned before due to high specificity and sensitivity, CT scan is gold standard for ureterolithiasis diagnosis, the alternate choice is ultrasound, which is cost-effective and provides accurate outcome like CT scan without releasing of harmful ionizing radiation.^13^ Patients with obstructing ureteral stone and urosepsis need to undergo stent or nephrostomy. The probability of reducing diagnostic dilemmas in an emergency will improve patient quality of care.

Our study of external validation diagnosis of ureteral stone by STONE score 16.66% in the low-risk group, 54.28% in moderate-risk group and 85.71% in high-risk group is similar to Internal validation of Original study conducted by Moore et al at Yale university school of medicine. Their values were 8.3–9.2 % for low risk, 51.3–51.6 % for moderate-risk, and 88.6–89.6 % for high-risk.

### Clinical Significance of Study

The present study supports the use of STONE score for initial evaluation of patients presenting with flank pain. The study also recommends that there is a need of modifications in original STONE score for countries where there is no black race.

### Limitations and strength of study

This present study has some limitations, one of these is small size of our studied population and the other is that it is single center study. Being single center is also a strength of study because in all patients STONE score was calculated by a single person (the principal investigator) who got enough information about STONE score before starting study. Secondly all of CT reports were also evaluated by single radiologist that minimized inter-observer variability in study results.

## CONCLUSION

Based on our study results, CT scan and ultrasonography are standard diagnostic tools for suspected ureterolithiasis but in emergency unit, use of STONE score to categorize the patient as low risk, moderate-risk and high-risk of ureteral stone can help the physician (clinician) to take decision either there is a need of further investigation or not.

### Author’s Contribution:

**AM:** Conceived, prepared the manuscript, and is responsible for integrity of study.

**SM:** designed the research methodology, supervised the research work, did review and gave final approval for publication.

**SY:** Helped in data collection and analyusis.

**MARB and AA:** Did data Analysis, did review and helped to finalize the manuscript.
